# Novel link prediction for large-scale miRNA-lncRNA interaction network in a bipartite graph

**DOI:** 10.1186/s12920-018-0429-8

**Published:** 2018-12-31

**Authors:** Zhi-An Huang, Yu-An Huang, Zhu-Hong You, Zexuan Zhu, Yiwen Sun

**Affiliations:** 1 College of Computer Science and Software Engineering, Shenzhen Universit1y, Shenzhen, 518060 China; 2 Department of Computer Science, City University of Hong Kong, Hong Kong, 999077 China; 3 Department of Computing, Hong Kong Polytechnic University, Hong Kong, 999077 China; 4 School of Medicine, Shenzhen University, Shenzhen, 518060 China

**Keywords:** miRNA-lncRNA interaction, ceRNA network, Expression profile, Collaborative filtering, Computational prediction

## Abstract

**Background:**

Current knowledge and data on miRNA-lncRNA interactions is still limited and little effort has been made to predict target lncRNAs of miRNAs. Accumulating evidences suggest that the interaction patterns between lncRNAs and miRNAs are closely related to relative expression level, forming a titration mechanism. It could provide an effective approach for characteristic feature extraction. In addition, using the coding non-coding co-expression network and sequence data could also help to measure the similarities among miRNAs and lncRNAs. By mathematically analyzing these types of similarities, we come up with two findings that (i) lncRNAs/miRNAs tend to collaboratively interact with miRNAs/lncRNAs of similar expression profiles, and vice versa, and (ii) those miRNAs interacting with a cluster of common target genes tend to jointly target at the common lncRNAs.

**Methods:**

In this work, we developed a novel group preference Bayesian collaborative filtering model called GBCF for picking up a top-*k* probability ranking list for an individual miRNA or lncRNA based on the known miRNA-lncRNA interaction network.

**Results:**

To evaluate the effectiveness of GBCF, leave-one-out and *k*-fold cross validations as well as a series of comparison experiments were carried out. GBCF achieved the values of area under ROC curve of 0.9193, 0.8354+/− 0.0079, 0.8615+/− 0.0078, and 0.8928+/− 0.0082 based on leave-one-out, 2-fold, 5-fold, and 10-fold cross validations respectively, demonstrating its reliability and robustness.

**Conclusions:**

GBCF could be used to select potential lncRNA targets of specific miRNAs and offer great insights for further researches on ceRNA regulation network.

**Electronic supplementary material:**

The online version of this article (10.1186/s12920-018-0429-8) contains supplementary material, which is available to authorized users.

## Background

The advent of next-generation sequencing has opened up new avenues to understand specific biomechanism from genome wide biomolecular interactions. The essential role of non-coding RNAs (ncRNAs) in biological process reveals that the transcriptional landscape of humans and other organisms is far more complicated than previously thought [1]. As the majority of transcripts expressed in mammals, ncRNAs can measure from around 22 nucleotides up to hundreds of kb. Specially, long non-coding RNA (lncRNA) is a loosely classified group of RNA transcripts (> 200 nucleotide bases) without apparent protein-coding function and can be discovered in any branch of life [2]. Increasing evidence has shown that lncRNAs can participate in various cellular processes including mRNA splicing, protein translation, cell growth/death through influencing chromatin modification, and cell differentiation and transcriptional complex targeting. Even though more than 58,000 human lncRNA genes have been identified, apart from few well-studied lncRNAs like XIST and HOTAIR, most of them are still poorly characterized due to the dynamic and complicated molecular mechanisms [3].

LncRNAs are involved in the pattern regulations of expressed proteins by a specific mechanism comprising a variety of biological interactions such as lncRNA-ncRNA, lncRNA-mRNA and lncRNA-protein interactions [4]. Therefore, the construction of inferred biological interaction network mediated by lncRNAs should be desirable to uncover the potential mechanisms and biological functions of lncRNAs. LncRNA, as a main type of competing endogenous RNAs (ceRNAs), can function as miRNA sponges having a lower regulatory effect of miRNA on mRNAs, i.e., miRNAs have an important influence in the molecular mechanisms of lncRNAs [1]. Previous works of human lncRNA function annotation were mainly based on the expression level between lncRNAs and protein-coding genes in diverse tissues [5, 6], but few functional annotations were explained according to the ceRNA network. Along with the knowledge accumulation on miRNA function for the past decade, miRNA-lncRNA interactions can provide new insights into understanding the complex functions of lncRNA.

The important influence of miRNA on lncRNA function, and the converse, is now gaining widespread attention [3, 7]. Numerous of studies have demonstrated that both miRNA and lncRNA get involved in pathological processes including diverse human disorders and diseases, and the regulation role of miRNA-lncRNA interactions in some human complex diseases have been systematically investigated [8]. For example, the miRNA-lncRNA regulatory networks in vascular diseases and cancers (e.g. gastric cancer and prostate cancer) have been well constructed and studied in [9–11]. The detailed understanding of the effects of miRNA-lncRNA-mediated interactions in pathophysiology could pave the way for drug toxicology, biomarker discovery and therapeutic approaches. However, the current knowledge of miRNA-lncRNA interactions identified by biological experiments is still limited.

In recent years, computational models have been extensively used for predicting bi-partite relationships (e.g. drug-target interactions [12–15], lncRNA-disease associations [16] and microbe-disease associations [17–19]). As an indispensable step to identify miRNA-target interactions, it is a common practice to develop computational prediction for refining the candidate list before further experimental validation [20, 21]. However, most existing miRNA-target inference algorithms were initially proposed for mRNA targets, and the inferences are therefore based on the statistical rules and nature of miRNA-mRNA interactions [22]. The common rules on which most existing miRNA-target prediction tools are based mainly come from four aspects conservation, seed match, free energy, and site accessibility, but some of them could even contradict with the nature of miRNA-lncRNA interactions [3]. For example, based on the observation that the miRNA seed regions of mRNA tend to have apparently higher conservation than the non-seed ones. A few previously proposed prediction approaches for miRNA-target interactions conduct the conservation analysis primarily concentrating on the regions in the 3’ UTR and the 5’ UTR of mRNA. However, lncRNAs have been found to demonstrate distinctly lower sequence conservation and faster evolution than mRNAs [3]. Moreover, the statistic rules on which the strategy of seed match is based are firstly arising from miRNA-mRNA interactions, and therefore not suitable for miRNA-lncRNA interaction prediction. There have been a number of computational prediction models proposed for lncRNA-RNA interaction via the simple calculation of the free energy of the potential binding sites [3]. For instance, LncTar was proposed to calculate the free energy served as the measurement of the stability of complementarity between lncRNAs and target RNAs [22]. Such sequence-based inference methods achieve successes in various applications, however they could be easily plagued by the high false positive rates [20]. In addition, there exist a few inherent characteristics distinguishing between lncRNAs and mRNAs. For example, unlike mRNAs, lncRNAs are more enriched and lowlier expressed in the nucleus. They are also shorter with fewer exons and have higher specificity of tissue distribution as well as reduced stability [3]. Most previously proposed miRNA-target inference tools fail to incorporate recent achievements of the understanding of miRNA-lncRNA interaction and could therefore not be effective for miRNA-lncRNA interaction inference.

Recent studies have provided insights into modeling the crosstalk among diverse types of ceRNAs including miRNAs and lncRNAs within the cell [23]. On top of the well-known factors such as miRNA response element (MRE) accessibility related to RNA-binding protein or secondary structure as well as subcellular localization, the expression profiling of lncRNA and miRNA is an important way to decipher the principles of ceRNA regulation networks [24]. Previous researches including small RNA (sRNA) regulation [25], protein-protein interactions [26–28], and miRNA-target threshold effects [29] suggest that miRNAs and lncRNAs serve as two key components of ceRNA network, and a titration mechanism helps to orchestrate their interaction with each other by forming threshold levels of effect. This titration mechanism is based on the basic postulate that limited number of available miRNA could contribute to the inactiveness of lncRNA, conversely the abundant miRNA molecules could result in the completely repressed lncRNA, so the optimal miRNA-lncRNA cross-regulation emerges and sustains at a near-equimolar equilibrium [24]. In other words, RNA dosage for cross-regulation in ceRNA network is particularly critical. It is worth to note that a kinetic mathematical model [24] under such considerations was proposed for the inference of ceRNA interactions mediated via phosphatase and tensin homolog (PTEN). However, all the factors used by this model, such as degradation and transcription rates for association and dissociation of miRNA/ceRNAs complexes [24], are too difficult to be surveyed for most of miRNAs and lncRNAs. Therefore, it is not feasible to extensively use this kinetic model for the inference of miRNA-lncRNA interactions. Increasing evidences [30, 31] demonstrated that lncRNAs are also presumably co-regulated in expression networks, and multiple lncRNAs could involve in the biological regulation processes by synergistically interacting particular miRNA clusters. Accordingly, the expression pattern of lncRNA-lncRNA synergistic network has recently attracted increasing attention.

In this work, we develop a group-preference *Bayesian* collaborative filtering model called GBCF to pick up a top-*k* probability ranking list for an individual miRNA or lncRNA based on the known miRNA-lncRNA interaction network derived from lncRNASNP database. Since the known miRNA-lncRNA interactions in the lncRNASNP database are all positive, the negative samples are relatively hard to be collected. This prediction task is actually a semi-supervised one only treating the known interactions as positive samples. The semi-supervised prediction task can properly utilize enough side information beneficial for the prediction performance. Particularly, we first propose the local scoring scheme to alleviate the prediction preference caused by the disproportion of the known miRNA-lncRNA interaction network. In this scoring system, we implemented both leave-one-out cross validation (LOOCV) and *k*-fold cross validation to evaluate the prediction performance of the proposed model. The experimental result demonstrated that GBCF obtain the reliable prediction performance and achieve the higher AUC (area under ROC curve) of 0.9193 compared with a few representative classical classifiers and the state-of-the-art model EPLMI [32]. GBCF obtained the average AUCs of 0.8354+/− 0.0079, 0.8615+/− 0.0078 and 0.8928+/− 0.0082 in the frameworks of 2-fold, 5-fold and 10-fold cross validations, respectively. To better describe the similarities among miRNAs and lncRNAs, we leveraged three diverse types of biological information, i.e., expression profile, coding-non-coding co-expression networks and sequence data. Using a series of 5-fold cross validations and correlation analysis of RNA clusters, the experimental comparison demonstrated that the miRNA and lncRNA similarity should be measured by the biological function-based and expression profile-based correlations, respectively.

## Results

### The experiment result in cross validations

Using LOOCV, we compared GBCF with a few classical classifiers including [33–36] as well as the state-of-the-art model EPLMI [30] as baseline. Note that, all the compared models were built on the same information source as GBCF. EPLMI is a two-way diffusion model first proposed for the prediction of large-scale miRNA-lncRNA interactions. Unlike GBCF, EPLMI adopts a global scoring scheme to rank the most potential novel miRNA-lncRNA interactions among all unobserved samples. We also tried to explore the potential of these classical classifiers from different perspectives. For example, Katz can be categorized as the network-based measurement method by calculating the nodes’ similarity in a bipartite graph. Singular-value decomposition (SVD) is used to decompose the known interaction network into three relatively smaller matrices for construction of probability matrix. Latent factor model (LFM) aims to explain observed associations in terms of two latent factors (also called hidden variables), which are iteratively optimized for matrix product as probability matrix. Since GBCF model adopts a specific group-preference Bayesian collaborative filtering (CF) technique, we also compared it with typical lncRNA-based and miRNA-based CF models, respectively.

**Fig. 1:**
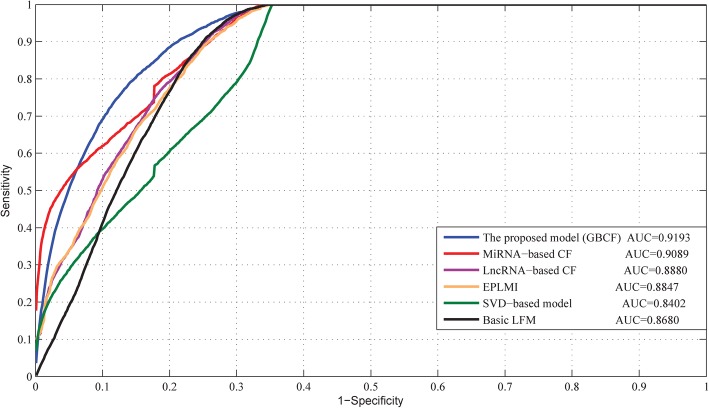
- The comparison results between GBCF and four classical classifiers as well as the competitor EPLMI model in terms of LOOCV

The performance comparison via LOOCV is shown in Fig. 1. Among these models, GBCF achieves the best prediction performance with the highest AUC value of 0.9193. The miRNA-based CF, lncRNA-based CF, EPLMI, SVD-based model and basic LFM obtain the AUC values of 0.9089, 0.8880, 0,8847, 0.8402 and 0.8680 respectively. It is noteworthy that the CF-based models seems to perform better than others do. This phenomenon could be attributed to their capability of automatic collecting extrinsic preferences from other RNAs. Although EPLMI model still maintain reasonable prediction accuracy, the local ranking scheme limit its performance to a certain extent. GBCF is developed from the previous approach of the recommended system, it is more efficient to deal with the sparse dataset than EPLMI. In a word, the LOOCV results demonstrate the reliability of GBCF.

Insufficient training samples would greatly affect the prediction accuracy (sparsity = 2.49%). To evaluate the performance of GBCF in terms of diverse sparsity, 2-fold, 5-fold and 10-fold cross validations were conducted, respectively. As shown in Table 1, GBCF model achieves the average AUCs of 0.8354+/− 0.0079 when the number of training samples drops to a half. In addition, the result suggests that GBCF model shows a strong robustness to different level of training data sparsities. We also used 5-fold cross validation to assess the performance of GBCF with lncRNA-based group preference instead. The average AUCs of 0.8612+/− 0.0080 obtained suggest that miRNA- and lncRNA- based group preferences contribute equally to the prediction performance of GBCF. Considering the complex competition mechanisms in ceRNA network and the lack of investigation into the competition patterns for sequestering miRNAs, we provided the top-50 ranking lists of candidate target lncRNAs for each type of miRNA with the corresponding prediction scores by using miRNA-based group preference, respectively (publicly available in Additional file 1). It is anticipated that these prediction results could shed light on deciphering the clues of ceRNA regulation networks.

**Table 1:** The experiment result of *k*-fold cross validation

*K*-fold	2	5	10
Average AUCs	0.8354+/−0.0079	0.8615+/−0.0078	0.8928+/− 0.0082

### The performance evaluation with different types of RNA similarity

In this subsection, we explore the effective measurement of different RNA similarities, i.e. sequence-based similarity, expression profile-based similarity and biological function-based similarity derived from RNA-target gene interactions. To evaluate the prediction performance with different types of RNA similarity, 5-fold cross validation was used in this comparison experiments (see Table 2). When fairly evaluating the usefulness of similarity for one type of RNA, another type of RNA was assigned the best similarity, i.e., the expression profile-based similarity for lncRNA and biological function-based similarity for miRNA.

**Table 2:** To evaluate the usefulness of diverse types of RNA similarity, 5-fold cross validation was implemented on GBCF model

Types of similarity	Average AUCs
lncRNA	
Sequence-based	0.8084+/−0.0080
Expression profile-based	0.8615+/−0.0078
Biological function-based	0.8219+/−0.0081
miRNA	
Sequence-based	0.7729+/−0.0078
Expression profile-based	0.8382+/−0.0081
Biological function-based	0.8615+/−0.0078

With regard to lncRNA, GBCF model yields the highest average AUCs 0.8615+/− 0.0078 using the expression profile-based similarity. In addition, GBCF obtains lower average AUCs of 0.8084+/− 0.0080 and 0.8219+/− 0.0081 based on the sequence- and biological function-based similarities, respectively. Since there is a large difference in the lengths of the lncRNAs, we concentrated the investigation in the range 73 to 59,462 bp. Pairwise global alignment tends to fail the measurement of sequence similarities among lncRNAs via their nucleotide bases. Moreover, unlike miRNAs, lncRNAs could play different biological roles in ceRNA network. For example, miRNAs tend to sequestered via small-binding sites in lncRNAs. The known annotations based on the coding-non-coding co-expression network could not comprehensively describe how biologically similar the regulation mechanisms of two lncRNAs could be. In a word, this result demonstrates that expression profiling could be a promising marker to characterize lncRNA similarity.

As for miRNA similarities, the comparison results demonstrate that they make different contribution to the performance of GBCF. The result in Table 2 shows that miRNA sequence-, expression profile- and biological function-based similarities yield average AUCs of 0.7729+/− 0.0078, 0.8382+/− 0.0081 and 0.8615+/− 0.0078, respectively. The AA index as a local similarity-based method could better explore the implicit topological information among miRNAs from the network of miRNA-target gene interactions. Therefore, the biological function-based similarity with the best average AUCs was chosen as the miRNA similarity measurement. We also investigated the prediction performance of GBCF without any similarity but known miRNA-lncRNA interactions as a baseline test. In this case, GBCF achieves average AUCs of 0.6840+/− 0.0116 also in 5-fold cross validation.

### Similarity analysis of miRNA and lncRNA clusters between observed and unobserved miRNA-lncRNA interactions

**Fig. 2:**
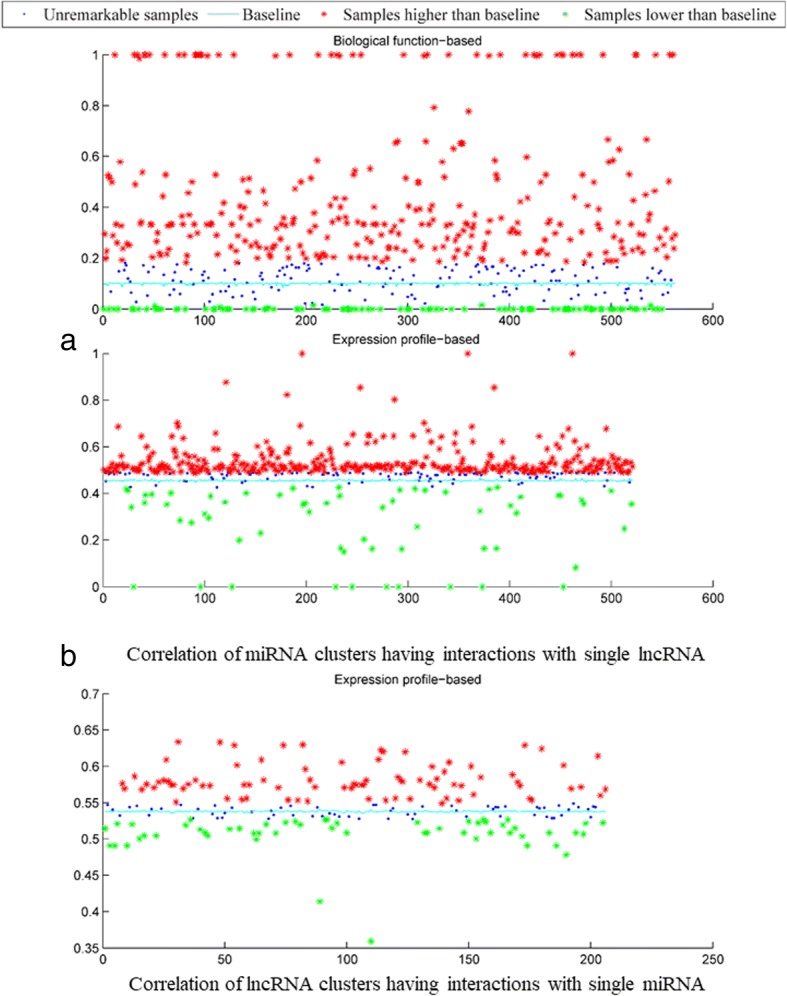
- Similarity correlation analysis

To further analyze the correlation of utilized RNA similarities between observed and unobserved miRNA-lncRNA interactions and evaluate the effectiveness of GBCF, we compared the differences in miRNA/lncRNA clusters interacting with single lncRNA/miRNA based on the known miRNA-lncRNA interaction network. For example, given the miRNA clusters interacting more than two lncRNAs, lncRNAs were divided into two groups: (i) the observed miRNA group and (ii) the unobserved miRNA group depending on whether they were found to interact with the miRNA. Then we used the average Pearson correlation coefficient (PCC) to measure the difference for each of those two lncRNA group. The average PCC of the unobserved group for each lncRNA served as the baseline of the comparison. LncRNA clusters also undertook the same procedure. To give a clear description, the function-based similarity of miRNA and expression profile-based similarity of both miRNA and lncRNA are representatively illustrated in Fig. 2. The comparison result is shown in Table 3. The remarkable samples with average PCC significantly higher or lower than the baseline (i.e., 0.3 times of the standard deviations of the observed RNA groups) are highlighted. There were 42.3% of lncRNA expression profiles unavailable in our dataset, and the investigated miRNAs had more opportunity to interact with lncRNAs (approximately 19 types of lncRNA for a miRNA). Under this condition, we analyzed the correlation of lncRNA clusters interacting with single miRNA based on expression profile and focused on the 206 well-studied miRNAs that have been identified to interact with more than 5 lncRNAs for more reliable conclusions.

**Table 3:** The data statistics of comparison results

miRNA			
Similarity	Function	Expression	Sequence
Invalid RNA	1.1%	16.4%	0
Higher than baselines	72.29%	83.50%	51.78%
lncRNA			
Similarity	Function	Expression	Sequence
Invalid RNA	66.2%	42.3%	1.3%
Higher than baselines	56.13%	59.22%	89.36%

With respect to miRNA, we found that most miRNA clusters sharing more similarity (average PCC higher than baselines) tend to interact with single lncRNA except for the sequence-based similarity, which is easily plagued by the relatively high false positive rates. Those RNAs which cannot be mapped into corresponding datasets could be considered as invalid. After excluding the invalid miRNA IDs, 72.29% (407/563) of miRNA clusters were found to be higher than the baselines based on the biological function-based similarity derived from the miRNA-target gene interaction network. For those 563 types of lncRNAs, the observed miRNA groups yield an average PCC of 0.2787, which is significantly higher than the average baseline PCC of 0.0994. This result suggests that the miRNAs interacting with a cluster of common target genes could jointly target common biological processes and therefore share more functional similarity. Apart from the miRNA biological function-based similarity, it is interesting to note that most correlations of expression profile-based similarity tend to approach the baseline. In a word, those miRNA clusters interacting with lncRNAs are likely to have similar expression patterns. Based on the miRNA expression profile-based similarity, the average PCC of 83.50% (435/521) of the miRNA clusters are higher than the baselines (0.4551) achieving the value of 0.4947. This result demonstrates that, although containing a number of invalid miRNA IDs (16.4%), miRNA expression profile-based similarity indeed can reflect the regulation mechanisms in miRNA-lncRNA interaction network and therefore deserves more future investigation. We can see that the predictive power of GBCF is not be affected for RNA with low similarity to known miRNAs/lncRNAs. As shown in Fig. 2a, the average PCCs of the baselines are 0.4551 and 0.0994 based on the miRNA expression profile-based similarity and miRNA biological function-based similarity, respectively. Obviously, the value of the expression profile-based similarity is significantly higher than the biological function-based. However, GBCF achieves better prediction performance using the miRNA biological function-based similarity. Therefore, the low RNA similarity to known miRNAs/lncRNAs dose not interfere with the predictive power of GBCF.

As for lncRNA expression profile-based similarities, after excluding the invalid lncRNA IDs, 59.22% (122/206) of lncRNA clusters were shown to share more similarity on the observed miRNA-lncRNA network. For those 206 well-studied miRNAs, the average PCC of the observed lncRNA clusters is 0.5476, which is slightly higher than the average baseline PCC of 0.5378. Note that approximately 71.3% (87/122) remarkable samples obtain the average PCC higher than the baselines and above the threshold range. The result also reflects the fact that expression profiling could be a promising feature to measure the correlation of lncRNA clusters with their miRNA-mediated principles of regulation. 22 types of lncRNA expression level we collected could not be sufficient to effectively detect the expression patterns of an individual lncRNA. Certainly, there is a huge potential for lncRNA expression profile-based similarity.

Finally, we evaluated the other two types of lncRNA similarities in the same way. As a result, 56.13 and 89.36% of the lncRNAs have the average PCC higher than the baselines. Sequence-based lncRNA similarity cannot be used to differentiate the types of lncRNA. Moreover, the common parts shared among lncRNAs are only a small portion of their total lengths, so the baselines of lncRNA sequence similarity are relatively low. The pairwise global alignment fails to precisely measure the sequence similarities among lncRNAs via their nucleotide bases.

## Discussions

The study leads to the following findings. First, the similarities among miRNAs/lncRNAs derived from expression profile and coding-non-coding co-expression networks are effective to be representative measurements. Second, group preference *Bayesian* collaborative filtering technique shows a strong capability to synergistically incorporate extrinsically implicit topological information in ceRNA regulation network. Finally, the local scoring system proposed in this domain is useful to alleviate the prediction preference brought by the disproportionate learning samples in the known miRNA-lncRNA interaction network. However, we also noticed that a few limitations indeed affected the prediction performance of GBCF. For example, it is insufficient to collect the lncRNA expression levels in 16 different human tissues and 8 cell lines. More remarkable features should be gathered to improve the reliability of lncRNA expression profile-based similarity measurement. There are many parameters to tune, which means that it is difficult to optimize the prediction performance in short term.

Based on GBCF, we can carry out the further research from two viewpoints. First, the indirect lncRNA-lncRNA interactions in ceRNA network could be inferred. It has been found that indirect lncRNA-lncRNA interactions in ceRNA network could be considered as the third transcripts supporting the crosstalk between two ceRNAs. As in the correlation analysis of lncRNA similarity between observed and unobserved miRNA-lncRNA interactions, the lncRNA clusters interacting with single miRNA with high scores tend to have frequently an indirect interaction. Second, GBCF can be used to measure different competitive status of how the lncRNAs are competitive to sequester a certain type of miRNA. As competing ceRNAs, target lncRNAs could coexist in ceRNA network where the quantity and effect of their MREs may not be consistent. In LOOCV and *k*-fold cross validation, the known miRNA-lncRNA interactions ranked in top list could play a more biologically significant role in ceRNA interaction network than others. The lncRNAs in such kind of interactions would have a priority to interact with miRNAs for maintenance of biological stability in ceRNA network. In other words, for the known miRNA-lncRNA interactions ranked in top list, the lncRNAs assigned with higher scores by GBCF are likely to interact with miRNAs more competitively.

## Conclusions

Enormous evidences focus on the miRNA-lncRNA interactions to explore the potential regulation mechanisms in ceRNA network. It is still insufficient to promote the development of this domain given current knowledge and data regarding to the observed miRNA-lncRNA interactions. Little effort has been devoted to the large-scale prediction of miRNA-lncRNA interactions except some sequence-based prediction methods mainly focusing on predicting target genes/mRNA for a miRNA. We came up with three different measurements for RNA similarity from three diverse types of biological information, namely expression profile, coding-non-coding co-expression networks and sequence data, respectively. Through a series of 5-fold cross validation and correlation analysis of RNA clusters in observed samples, the experimental results suggest that (i) lncRNAs/miRNAs tend to collaboratively interact with miRNAs/lncRNAs of similar expression profiles, and vice versa, and (ii) miRNAs interacting with a cluster of common target genes tend to jointly target common lncRNAs. We utilized group preference *Bayesian* collaborative filtering technique for a large-scale prediction of miRNA-lncRNA interactions. LOOCV and 5-fold cross validation were used to demonstrate the usefulness of the proposed model through the comparison with a few classical classifiers and the state-of-the-art model EPLMI.

## Methods

### Materials

Data used for construction of the known miRNA-lncRNA interaction network were taken from the lncRNASNP database (the February 2017 version), which is publicly available at http://bioinfo.life.hust.edu.cn/lncRNASNP [37]. All curated records were confirmed via laboratory examination with research literatures. Based on 108 CLIP-Seq datasets, lncRNASNP provides 8091 pairwise interactions. After excluding the repetitive entries, we collected totally 5348 pairs of interactions (denoted as *P*_ml_). These interactions involve 275 (denoted as *nm*) diverse types of miRNAs and 780 (denoted as *nl*) diverse types of lncRNAs.

To calculate the similarities among lncRNAs from different perspectives, three types of biological information were gathered from various databases. First, the expression profile data and inferred functional annotations of lncRNAs were accessible from the NONCODE database (http://www.noncode.org/) [38],. We obtained the expression profiles for 450 of the lncRNAs and the functional annotations for 264 of the lncRNAs after mapping the NONCODE IDs into the names of the investigated lncRNA. Second, the gathered expression profiles for each type of lncRNAs with 22 attributes, respectively representing the expression level of 16 different human tissues and 8 cell lines. The putative functional annotations for each lncRNA genes refer to the top-10 most possible biological functions, which are inferred by lnc-GFP method [39] based on a coding-non-coding co-expression network. Finally, the sequence data of each lncRNA were downloaded from LNCipedia database (https://lncipedia.org/) [2].

Similarly, the three same types of biological information were collected for measuring the similarities among miRNAs. miRTarBase (http://miRTarBase.mbc.nctu.edu.tw) [40] curates a large number of miRNA and multi-gene interactions. We successfully converted the miRTarBase IDs into the names of 272 investigated miRNAs. microRNA.org database [41] provides the expression profile data of miRNAs, 230 of which were found to be matched. The expression profile of each miRNA has 172 attributes describing the expression levels of 172 various tissues and cell lines in human body. miRBase database (http://www.mirbase.org/index.shtml) [42, 43] offers us the sequence data of mature miRNAs.

### The sequence-based similarity of RNAs

Based on the obtained lncRNA/miRNA sequence data, the Needleman-Wunsch pairwise sequence alignment was implemented to measure the sequence similarity of lncRNAs and miRNAs by leveraging the package of pairwise2 in *Biopython* [44]. In this work, the identification score, gap-open penalty and gap-open extending penalty were set to 2, − 0.5 and less 0.1, respectively. It need to note that, it is unnecessary to compare miRNA sequence-based similarity and lncRNA sequence-based similarity, since the sequence-based similarity is calculated among the same type of RNA and then normalized as a weight from 0 to 1. In this regard, it has no influence to the final prediction score.

### The expression profile-based similarity of RNAs

The expression pattern could be an important ingredient for RNA similarity measurement. Namely, the more biologically possible lncRNAs/miRNAs could have the more consistent expression levels in human tissues and cell lines. Therefore we simply used PCC to calculate such kind of RNA similarity based on the collected expression profiles as follow:

1 $$ \mathrm{ES}\left(\mathrm{i},\mathrm{j}\right)=\frac{\sum_{k=1}^N\left({e}_{ik}-\overline{e_i}\right)\left({e}_{jk}-\overline{e_j}\right)}{\sqrt{\sum_{k=1}^N{\left({e}_{ik}-\overline{e_i}\right)}^2{\sum}_{k=1}^N{\left({e}_{jk}-\overline{e_j}\right)}^2}} $$

where *i* and *j* refer to two same-type RNAs. *e*_*ik*_ represents the *kth* attribute of the expression profile of RNA *i*. Parameter *N* is the number of attributes of the expression profiles (i.e. *N* = 22 for lncRNAs, and *N* = 172 for miRNAs). The higher *ES(i,j)* is, RNAs *i* and *j* are more similarly expressed in general.

### The biological function-based similarity of RNAs

Based on the hypothesis that lncRNAs/miRNAs sharing more similar regulation mechanisms and features tend to have interactions with a cluster of target genes, we compute such the correlation of how a pair of RNAs is functionally similar based on the data of RNA-target gene interactions. According to Cubero’s work [45], local similarity-based methods have been extensively applied and shown a very competitive prediction accuracy against more complex approahces. To better exploit the implicit information from the topological network structure, four typical methods were chosen for the functional similarity measurement, i.e. Common Neighbors (CN), the Adamic-Adar (AA) Index, the Jaccard (JA) Index and the Salton (SA) Index [45]. Given two RNAs *i* and *j* within the same type, these four methods can be described as follows:

2 $$ \mathrm{CN}\left(\mathrm{i},\mathrm{j}\right)=\left|{\Gamma}_i\cap {\Gamma}_j\right| $$

3 $$ \mathrm{AA}\left(\mathrm{i},\mathrm{j}\right)=\sum \limits_{z\in {\Gamma}_i\cap {\Gamma}_j}\frac{1}{\mathit{\log}\left|{\Gamma}_z\right|} $$

4 $$ \mathrm{JA}\left(\mathrm{i},\mathrm{j}\right)=\frac{\left|{\Gamma}_i\cap {\Gamma}_j\right|}{\left|{\Gamma}_i\cup {\Gamma}_j\right|} $$

5 $$ \mathrm{SA}\left(\mathrm{i},\mathrm{j}\right)=\frac{\left|{\Gamma}_i\cap {\Gamma}_j\right|}{\sqrt{\left|{\Gamma}_i\right|\left|{\Gamma}_i\right|}} $$

here the set of nodes (target genes) connected through an edge to a RNA *i* is called the neighborhood of *i* and is denoted as Γ_*i*_. After 5-fold cross validation, the *AA* Index and the *SA* Index achieved the best prediction accuracy for miRNAs and lncRNAs, respectively, and therefore were respectively used as their functional similarity.

### Group-based Bayesian collaborative filtering computational model

**Fig. 3:**
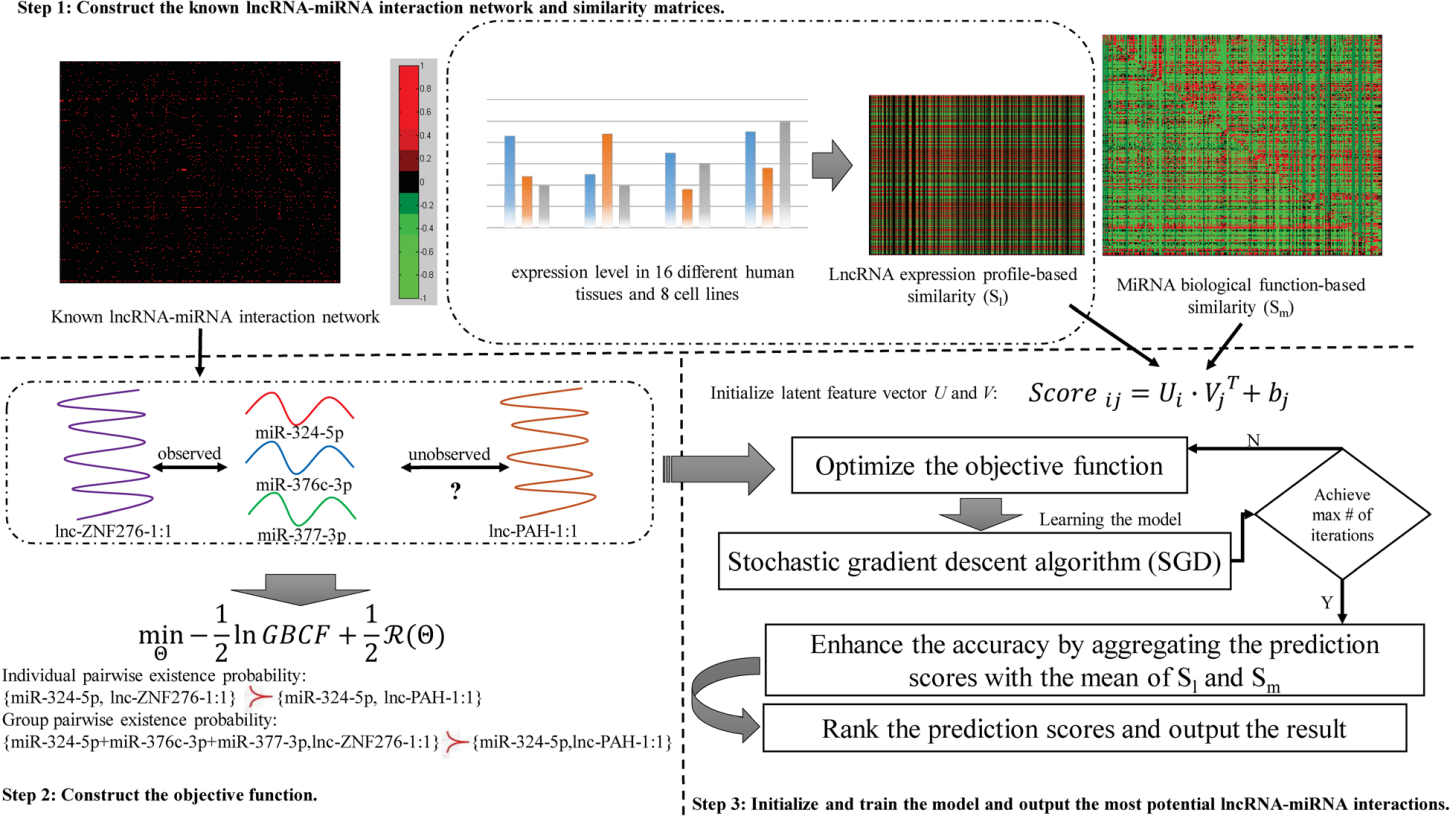
- The flowchart of GBCF

Inspired by Pan’s work [46], especially the injection of richer interactions via group preference, we explored a novel computational model called GBCF for ceRNA interaction inference based on the lncRNA-lncRNA similarity (denoted as *S*_*l*_), miRNA-miRNA similarity (denoted as *S*_*m*_) and known miRNA-lncRNA interaction network (see Fig. 3). Due to the absence of the negative miRNA-lncRNA interactions, i.e., pairs of miRNA and lncRNA have been experimentally confirmed having no interactions, the prioritization for potential candidates is in the basis of *Bayesian* inference by treating that the unobserved interactions (i, j) are less likely to exist than the observed ones (i, k). Here we use (i, k) ≻ (i, j) to denote that miRNA *i* is more likely to have interactions with lncRNA *k* than lncRNA *j*. The result of 5-fold cross validation suggests that *S*_*l*_ should be expression profile-based while *S*_*m*_ should be replaced by the biological function-based.

At the beginning of the prediction process of GBCF, *S*_*l*_ and *S*_*m*_ are fed to the information source for the construction of the latent feature vector *U(lncRNA)* and *V(miRNA)* as initialization parameters, respectively, i.e. U ∈ *ℝ*^1 ∗ *nm*^, *V* ∈ *ℝ*^1 ∗ *nl*^. To describe the method more clearly, in this case, we impose the group preference on miRNA uniformly. In this way, the group preference can be considered as an overall preference score of a group of miRNAs on a lncRNA. For example, given a group of miRNAs $\mathcal{G}$ and a lncRNA *j*, the overall group preference score of $\mathcal{G}$ on *j* can be calculated from individual preferences as ${\mathrm{Score}}_{\mathcal{G}j}=\frac{1}{\left|\mathcal{G}\right|}{\sum}_{i\in \mathcal{G}}{Score}_{ij}$. ${\mathcal{M}}^{tr}={\left\{m\right\}}_{m=1}^{nm}$ and ${\mathcal{L}}^{tr}={\left\{l\right\}}_{l=1}^{nl}$ denote the training sets of miRNAs and lncRNAs, respectively. $\mathrm{j}\in {\mathcal{L}}_i^{tr}$ means the miRNA-lncRNA pair (i, j) is observed while $\mathrm{k}\in {\mathcal{L}}^{tr}\backslash {\mathcal{L}}_i^{tr}$ means (i, k) is not observed. Empirically, if $\mathrm{j}\in {\mathcal{L}}_i^{tr}$ and $\mathrm{k}\in {\mathcal{L}}^{tr}\backslash {\mathcal{L}}_i^{tr}$, the group pairwise preference can be estimated conceptually, $\left(\mathcal{G},\mathrm{j}\right)\succ \left(\mathcal{G},\mathrm{k}\right)$ where $\mathrm{i}\in \mathcal{G}$ and $\mathcal{G}\subseteq {\mathcal{M}}_j^{tr}$. To precisely learn the unified effect of individual preference and group preference, we linearly combined them as follows:

6 $$ \left(\mathcal{G},\mathrm{j}\right)+\left(\mathrm{i},\mathrm{j}\right)\succ \left(\mathrm{i},\mathrm{k}\right)\  or\ {Score}_{\mathcal{G} ij}>{Score}_{ik} $$

where ${Score}_{\mathcal{G} ij}=\rho {Score}_{\mathcal{G}j}+\left(1-\rho \right){Score}_{ij}$, and *ρ* is a tradeoff parameter fusing such two kinds of preferences, ranging from 0 to 1 (*ρ*=0.5 in this study). In this way, a novel index called *group Bayesian collaborative filtering* (GBCF) ranking for miRNA *i* is denoted as follows:

7 $$ \mathrm{GBCF}\left(\mathrm{i}\right)={\prod}_{j\in {\mathcal{L}}_i^{tr}}{\prod}_{k\in {\mathcal{L}}^{tr}\operatorname{}{\mathcal{L}}_i^{tr}}\Pr \left({Score}_{\mathcal{G} ij}>{Score}_{ik}\right)\left[1-\Pr \Big({Score}_{ik}>{Score}_{\mathcal{G} ij}\right] $$

Given two miRNAs *i* and *t*, the joint likelihood could be simply approximated by the multiplication operation like GBCF(i, t) ≈ GBCF(i)GBCF(t). As such, the correlation between *i* and *t* is introduced via the miRNA group $\mathcal{G}$. Specifically, these two miRNA groups $\mathcal{G}\left(\mathrm{i},\mathrm{j}\right)\subseteq {\mathcal{M}}_j^{tr}$ and $\mathcal{G}\left(\mathrm{t},\mathrm{j}\right)\subseteq {\mathcal{M}}_j^{tr}$ may be overlapped, namely $\mathcal{G}\left(\mathrm{i},\mathrm{j}\right)\cap \mathcal{G}\left(\mathrm{t},\mathrm{j}\right)\ne \varnothing$. The overall likelihood is estimated for all miRNAs and all lncRNAs as follows:

8 $$ \mathrm{GBCF}={\prod}_{i\in {\mathcal{M}}^{tr}}{\prod}_{\mathrm{j}\in {\mathcal{L}}_i^{tr}}{\prod}_{k\in {\mathcal{L}}^{tr}\operatorname{}{\mathcal{L}}_i^{tr}}\Pr \left({Score}_{\mathcal{G} ij}>{Score}_{ik}\right)\left[1-\Pr \left({Score}_{ik}>{Score}_{\mathcal{G} ij}\right)\right] $$

where$\mathcal{G}\subseteq {\mathcal{M}}_j^{tr}$. Based on the previous work [47], $\upsigma \left({Score}_{\mathcal{G} ij}-{Score}_{ik}\right)=\frac{1}{1+\exp \left(-{Score}_{\mathcal{G} ij}+{Score}_{ik}\right)}$is used to approximate the probability $\Pr \left({Score}_{\mathcal{G} ij}>{Score}_{ik}\right)$, and finally have $\Pr \left({Score}_{\mathcal{G} ij}>{Score}_{ik}\right)\left[1-\Pr \left({Score}_{ik}>{Score}_{\mathcal{G} ij}\right)\right]={\sigma}^2\left({Score}_{\mathcal{G} ij}-{Score}_{ik}\right)$. The objective function of GBCF could be reached as follows:

9 $$ \underset{\Theta}{\min }-\frac{1}{2}\ln GBCF+\frac{1}{2}\mathcal{R}\left(\Theta \right) $$

whereΘ = {*U*, *V*, *b*_*j*_*ϵℝ*} is a set of model parameters to be learned. $\mathcal{R}\left(\Theta \right)={\prod}_{i\in {\mathcal{M}}^{tr}}{\prod}_{j\in {\mathcal{L}}_i^{tr}}{\prod}_{k\in {\mathcal{L}}^{tr}\operatorname{}{\mathcal{L}}_i^{tr}}\left[{\alpha}_m{\sum}_{t\in \mathcal{G}}{\left\Vert {U}_t\right\Vert}^2+{\alpha}_l{\left\Vert {V}_j\right\Vert}^2+{\alpha}_l{\left\Vert {V}_k\right\Vert}^2+{\beta}_l{\left\Vert {b}_j\right\Vert}^2+{\beta}_l{\left\Vert {b}_k\right\Vert}^2\right]$ is the regularization term to avoid overfitting, where *α*_*m*_, *α*_*l*_ and *β*_*l*_ are regulation weights ranging from 0.001 to 0.1. The objective function in Eq. (9) can be rewritten as:

10 $$ \mathrm{f}\left(\mathcal{G},\mathrm{i},\mathrm{j},\mathrm{k}\right)=-\ln \left({Score}_{\mathcal{G} ij}-{Score}_{ik}\right)+\frac{\alpha_m}{2}{\sum}_{t\in \mathcal{G}}{\left\Vert {U}_t\right\Vert}^2+\frac{\alpha_l}{2}{\left\Vert {V}_j\right\Vert}^2+\frac{\alpha_l}{2}{\left\Vert {V}_k\right\Vert}^2+\frac{\beta_l}{2}{\left\Vert {b}_j\right\Vert}^2+\frac{\beta_l}{2}{\left\Vert {b}_k\right\Vert}^2=\ln \left[1+\exp \left(-{Score}_{\mathcal{G} ij; ik}\right)\right]+\frac{\alpha_m}{2}{\sum}_{t\in \mathcal{G}}{\left\Vert {U}_t\right\Vert}^2+\frac{\alpha_l}{2}{\left\Vert {V}_j\right\Vert}^2+\frac{\alpha_l}{2}{\left\Vert {V}_k\right\Vert}^2+\frac{\beta_l}{2}{\left\Vert {b}_j\right\Vert}^2+\frac{\beta_l}{2}{\left\Vert {b}_k\right\Vert}^2 $$

We also use the stochastic gradient descent (SGD) algorithm to solve this optimization problem. The model parameters Θ can be updated as follows:

11 $$ \Theta =\Theta -\upgamma \frac{\partial f\left(\mathcal{G},i,j,k\right)}{\mathrm{\partial \Theta }} $$

where γ denotes the learning rate and is set to 0.1 in this study. The prediction score of miRNA *i* on lncRNA *j* is computed as ${Score}_{ij}={U}_i\bullet {V}_j^T+{b}_j$ each time until the model reaches the maximum number of iterations (default: 500). Using the 5-fold CV, we have tested the performance difference of GBCF with increasing maximum iteration (100, 300, 500 and 700). The result is tabulated in Table 4. We can see that GBCF achieved the highest average AUC of 0.8615+/− 0.0078 with 500 iterations. Running 700 iterations, the proposed model suffers from over-fitting and performance degradation. As such, the maximum iteration is empirically set to 500 by default. Note that a subset of miRNAs is randomly sampled as a miRNA group $\mathcal{G}$ before carrying out the SGD algorithm. To further enhance the prediction accuracy, for an unobserved pair miRNA *i* and lncRNA *j*, we aggregate *Score*_*ij*_ with the mean weight of *S*_*m*_(*i*^′^) and *S*_*l*_(*j*^′^), where ${i}^{\prime}\in {\mathcal{M}}_j^{tr}$ and ${j}^{\prime}\in {\mathcal{L}}_i^{tr}$ as follows.

12 $$ {Score}_{ij}+=\frac{\delta_m}{\left|{i}^{\prime}\right|}\sum \limits_{i^{\prime}\in {\mathcal{M}}_j^{tr}}{S}_m\left(i,{i}^{\prime}\right)+\frac{\delta_l}{\left|{j}^{\prime}\right|}\sum \limits_{j^{\prime}\in {\mathcal{L}}_i^{tr}}{S}_l\left(j,{j}^{\prime}\right) $$

where parameters *δ*_*m*_ and *δ*_*l*_ regulate the tradeoff of *S*_*m*_ and *S*_*l*_ respectively (*δ*_*m*_=*δ*_*l*_=1). The final *Score*_*ij*_ represents the existence probability of the unobserved miRNA-lncRNA pair. The pseudo-code of the proposed model is described in **Algorithm 1**. The model of GBCF is computationally efficient. The complexity of updating the objective function is $O\left(\left|\mathcal{G}\right|d\right)$, and the total time complexity of GBCF is $O\left( Tn\left|\mathcal{G}\right|d\right)$, where *T* is the maximum iteration, *n* is the number of miRNAs, $\left|\mathcal{G}\right|$ is the size of miRNA group and *d* is the total dimension number of latent feature vectors *U* and *V*.

**Table 4:** We used 5-fold cross validation to fine-tune the maximum number of iterations *T*

*T*	100	300	500	700
Average AUC	0.7800+/−0.0089	0.8333+/−0.0084	0.8615+/−0.0078	0.8503+/−0.0090

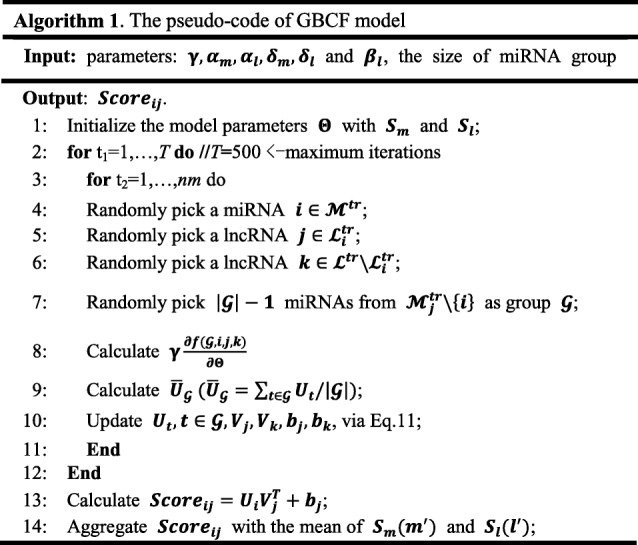



## Additional file

Additional file 1: The top-50 ranking lists of candidate target lncRNAs for each type of miRNA with the corresponding prediction scores by using miRNA-based group preference. (XLSX 410 kb)

## Abbreviations

ceRNAs: competing endogenous RNAs
CF: Collaborative filtering
CN: Common neighbors
LFM: Latent factor model
lncRNA: Long non-coding RNA
MRE: miRNA Response element
ncRNAs: Non-coding RNAs
PCC: Pearson correlation coefficient
PTEN: Phosphatase and tensin homolog
SGD: Stochastic gradient descent
sRNA: small RNA
SVD: Singular-value decomposition
